# Survival of *Ascaris *eggs and hygienic quality of human excreta in Vietnamese composting latrines

**DOI:** 10.1186/1476-069X-8-57

**Published:** 2009-12-16

**Authors:** Peter KM Jensen, Pham D Phuc, Flemming Konradsen, Lise T Klank, Anders Dalsgaard

**Affiliations:** 1 Department of International Health, Immunology and Microbiology, Faculty of Health, University of Copenhagen, Øster Farimagsgade 5. PO Box 2099.1014, Copenhagen K, Denmark; 2 National Institute of Hygiene and Epidemiology, Division of Enteric Infections, 1 Yersin Street, Hanoi, Vietnam; 3 National Veterinary Institute, Technical University of Denmark, Bülowsvej 27, DK-1790, Copenhagen V, Denmark; 4 Department of Veterinary Disease Biology, Faculty of Life Sciences, University of Copenhagen, Groennegaardsvej 15, DK-1870 Frederiksberg C, Denmark

## Abstract

**Background:**

For centuries farmers in Vietnam have fertilized their fields with human excreta collected directly from their household latrines. Contrary to the official guideline of six-month storage, the households usually only store human excreta for three to four months before use, since this is the length of time that farmers have available to produce fertilizer between two cropping seasons. This study aimed to investigate whether hygienically safe fertilizer could be produced in the latrines within this period of time.

**Methods:**

By inoculating eggs of the helminth parasite indicator *Ascaris suum *into heaps of human excreta, a die-off experiment was conducted under conditions similar to those commonly used in Vietnamese latrines. Half a ton of human excreta was divided into five heaps containing increasing concentrations of lime from 0% to 11%.

**Results:**

Regardless of the starting pH, which varied from 9.4 to 11.6, a >99% die-off of eggs was obtained after 105 to 117 days of storage for all lime concentrations and 97% of eggs were non-viable after 88 days of storage. The most critical parameter found to determine the die-off process was the amount of ammonia (urine) in the excreta which indicates that longer storage periods are needed for parasite egg die-off if urine is separated from the excreta.

**Conclusion:**

By inactivating >99% of all *A*. *suum *eggs in human excreta during a storage period of only three months the commonly used Double Vault Composting (DVC) latrine, in which urine is not separated, could therefore potentially provide a hygienic acceptable fertilizer.

## Background

The World Health Organization (WHO) estimates that two billion people are infected with helminths globally. Even with this staggering number of helminth infections, it is often given low priority by health authorities as helminthiasis is not associated with high mortality. However, helminth infections do result in high morbidity, reduced growth among children, and negatively impacts the learning capabilities of school children [1-4]. In China alone, the number of people infected with *Ascaris *is estimated to be more than half a billon [5]. Considering the high prevalence in many Asian countries, helminth infections can have a considerable impact on the health and economy of rural households. This is especially pronounced in Vietnam where the prevalence of helminth infections exceeds 80% in some rural areas in its northern and central regions, possibly related to the widespread use of excreta as a fertilizer input in agriculture [3,6-10].

In many parts of Vietnam and southern China human excreta is not perceived as a hazardous waste but as a "valuable fertilizer" in agriculture. This century old tradition is deeply rooted and widespread in agriculture and even today farmers will go to great lengths to access and use excreta. Some sanitation projects in Vietnam have failed partly because the promoted latrines did not accommodate the use of excreta for agriculture; latrines were either forced open or broken into by farmers who sought access to the otherwise sealed off excreta[11].

To limit any negative health impact of excreta use, the Vietnamese Ministry of Health has issued guidelines recommending a minimum six month composting/retention time of excreta in a latrine before use [9,12]. For the widely promoted and commonly used Double Vault Composting (DVC) latrine, this implies that the full vault should be sealed off for at least six months while the other vault is used. However, in central Vietnam around 80% of the farmers do not seem to follow these guidelines, since their agricultural practices demand sowing and fertilization of new crops with excreta every three to four months [13]. In Vietnam, the rural population typically adds kitchen ash and, occasionally, lime to the latrine following a toilet visit, primarily to reduce the moisture content, prevent bad odour and to combat flies [10]. These practices are likely to increase the inactivation of parasite eggs and other pathogens since dry conditions and increased pH (lime) negatively affect pathogen survival [14-16].

*Ascaris suum *eggs from pigs can be used as a model for the survival of *A. lumbricoides*, the roundworm that commonly infects humans in less developed countries [17]. Helminth parasite eggs are widely used as hygiene indicators as they are more resistant to environmental stress compared with viral, bacterial and other parasite pathogens. In the present study, *A. suum *eggs were chosen as a hygiene indicator as they are easily obtained and are less infective to humans than *A. lumbricoides*, although they may still complete part of their life cycle in humans. Since the viability and environmental resistance of *A. suum *eggs are considered to be equal to those of *A. lumbricoides *eggs and greater than those of other helminth eggs, the survival of *A. suum *eggs has successfully been used as a suitable indicator organism for the survival of *A. lumbricoides *and other helminth eggs (Holmquist & Stenström 2001). The aim of this study therefore was to use *A. suum *eggs to investigate whether the current practice of using of DVC latrines in Vietnam provides hygienically safe excreta fertilizer following a three to four month storage period. Furthermore, it was investigated whether lime applied in different quantities would increase pH and the parasite egg die-off and thus shorten the period required to produce hygienic excreta fertilizer.

## Methods

### Preparation of excreta for Ascaris egg survival experiments

In May 2005, approximately 500 kg of human excreta was collected from single and double vault composting latrines used by 25 households in a village in peri-urban Hanoi. The collected excreta consisted of both fresh excreta and excreta that had been stored for three to four months, a situation similar to that in a double vault latrine before one vault is sealed off for composting. The households supplying the excreta had added ash to it on a regular basis but not lime; one third of the households used a urine separation toilet, whereas the remaining households both urinated and defecated in the same vault.

The excreta was transported to a closed household yard that was protected from rain by a plastic sheet. All the excreta was mixed manually for an hour by shovel and five heaps containing between 35-53 kg excreta were made. The homogenization of the excreta from the different latrines was to minimize any bias that could result from some families having added more ash following defecation than others. It was decided to carry out the experiment in heaps of excreta rather than directly in vaults as it was impractical to inoculate helminth eggs (see below) in the middle of a heap inside the vault in such a way that the individual bags with eggs could be removed for analyses without disturbing the remaining parasite egg bags. To obtain different alkaline conditions, four heaps were added with 1%, 3%, 6% or 11% hydrated lime w/w (mass lime per mass wet excreta) and one heap without lime (control). The excreta heaps were then inoculated with helminth eggs (*Ascaris suum*) and their survival studied to assess the hygienic quality of the composted excreta.

### Preparation and inoculation of Ascaris eggs

Adult *A. suum *worms from the intestines of pigs were collected from a slaughterhouse in Hanoi city. The female worms were cut open and the last two centimetres of the bifurcation of the uterus, with mature eggs, was cut off. Pieces of the uterus were put in a test tube with tap water and a glass stick was used to press out the eggs from the uterus. The egg suspension was then passed through a sieve into another test tube to remove any large tissue fragments. The egg suspension was concentrated through centrifugation by 1000 rpm for five minutes. The supernatant was withdrawn and the pellet re-suspended in 0.05 M H_2_SO_4 _to suppress fungal and bacterial growth. A microscopic count (250-fold magnification) of 10 samples of 10 μl was used to determine the egg concentration. This egg stock solution, which contained approximately 2.2 × 10^4 ^eggs/ml, was stored in the refrigerator at 4-5°C up to three days before being inserted into the excreta heaps.

A rectangular piece (45 × 85 mm) of polyamide cloth (Monodur PA 31.5 N. Verseidag-Techfab GmbH, Geldern-Walbeck, Germany) with a pore-size of 20 μm (the size of the eggs are 45-70 × 35-50 μm) was used to produce the so-called tea bags that are widely used for helminth egg survival experiments [17]. Each bag contained approximately 20,000 *A. suum *eggs and a nylon fishing line was attached to allow for easy removal of the bags from the excreta heaps. The bags were kept in 0.1 M H_2_SO_4 _in a refrigerator for a day until brought to the field site and inserted into the heaps. The tea bags used for control samples were stored in 0.05 M H_2_SO_4 _in a refrigerator for up to six months.

Twenty four bags each with *A. suum *eggs were carefully placed in the centre of each of the five heaps. To capture biological variation and any impacts from variations in local environmental conditions inside the heap, two separate teabags were collected from each heap at each of the 12 sampling sessions. To further investigate the temperature effect on egg survival, four tea bags were placed in the top as well as in the bottom of the heap with no lime.

### Sampling and viability testing of Ascaris eggs

After 30 days of storage, two tea bags from each heap were removed by gently pulling the attached string. Subsequent sampling of tea bags was done every two weeks from May to November 2005. In addition to the sampling of the teabags inserted in the middle of the heap, the tea bags placed in the top and bottom of the heap with no lime added were sampled at 30-days intervals.

Viability testing of eggs was done according to earlier described methods on the day the tea bags were collected [17]. The tea bags were washed gently by filling them with distilled water and shaken in a way that concentrated the eggs at the bottom of the bags. The tea bags were opened with a pair of scissors and placed in separate petri dishes containing 10 ml 0.05 M H_2_SO_4 _with the fluid level marked on the side of the petri dish. Two petri dishes containing 0.05 M H_2_SO_4 _were prepared for each sampling time and kept as controls, with one dish containing free *Ascaris suum *eggs (2.2 × 10^4 ^eggs/ml) and another dish containing a closed tea bag with eggs, to assess the survival of the eggs in the solution as well as any impact of the tea bag on egg survival. These controls were used to calculate the percentage of viable eggs. The eggs and the fluid level were checked twice a week and the lids were taken off the petri dishes for 15 minutes twice a week to allow for adequate aeration of the eggs. If the fluid level was too low, distilled water was added. The egg viability was determined by comparing the percentage of viable eggs from the control with those of the sample. The eggs were incubated at room temperature (25-31°C) for four weeks before their survival were determined. By this time the viable eggs would have developed into larvae.

To determine the viability level of the eggs, i.e. whether a live larva had developed inside the eggs, they were carefully scraped, with a pipette, from the inside of the tea bags and placed in a drop of 0.05% methylene blue on a microscopy slide. A magnification of 10 and 45 times was used. The eggs were divided into three categories: those containing a dead larva (larva coloured blue), those containing a live larva and those that had not embryonated. Only whole eggs were counted. Viable eggs were defined as those containing a live larva. Where possible, a minimum of 100 eggs were counted and evaluated from each tea bag, thus no survivors counted would be equivalent to a 99% level of inactivation. If the eggs had not started to develop, only 20-30 eggs were counted in order to optimize the laboratory efficiency. The two teabags from each sampling heap were enumerated in parallel procedures and results were reported as the average of the two.

### Measurements of temperature

Seven Tiny talk^® ^temperature data loggers (Gemini Data Loggers (UK) Ltd. Chichester, United Kingdom.) in acid-resistant plastic bags were placed in the middle of the five heaps of excreta. To measure the possible different temperatures inside the excreta heap one heap also had temperature loggers placed in the top and bottom. Another two loggers were placed in the house and a shed next to where the heaps were placed to monitor the outdoor temperatures. The loggers were set to measure and log the temperature every four hours. At the end of the five month experiment the loggers were taken out of the heaps and the data uploaded to a computer.

### Moisture content, pH and nitrogen measurements

One cylinder (3 cm in diameter) of excreta sample was taken by driving a pipe down through the heap, as close to the centre as possible without hitting the tea bags. The sample was pushed out of the cylinder into a plastic bag and immediately transported to the National Institute of Soil and Fertilizer, Hanoi, for pH and moisture content analyses. Moisture content was measured on a monthly basis. Porcelain bowls were dried at 110°C in an oven for an hour, and weighed after cooling in a desiccator. Thereafter 10 g +/- 1 g of sample was placed in the bowl and weighed again. The bowl was then placed in a drying oven at 105°C ± 3°C overnight, then positioned in desiccators for cooling and thereafter weighed.

pH was measured at day zero and once every two weeks by adding 10 g of sample excreta to 50 ml of distilled water in a 100-ml glass beaker. The mixture was left to stand for 10 minutes and larger clumps of excreta were broken down manually with a glass rod. The suspension was then stirred for 10 minutes and thereafter a calibrated electrode (calibration at pH 7 and pH 10) was inserted in the suspension for pH measurement [18].

During the sampling period the heaps were measured for nitrogen content. Monthly, the pH samples were also analyzed for the content of Kjeldahl nitrogen (organic) and only once for the content of total nitrogen, as in the procedure described in [19].

## Results

**Figure 1:**
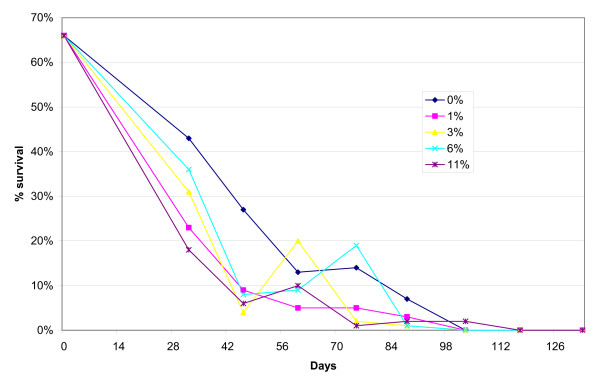
**The influence of storage time on survival of *Ascaris suum *eggs in heaps of human excreta under different alkaline conditions (% lime)**.

The 99% die-off time for *A. suum *eggs exposed to the different alkaline conditions ranged from 105 to 117 days (Figure 1). The survival rate of eggs used as controls was 63-70% throughout the experiment, with no difference between the control eggs inside the bags and the ones placed in petri dishes (results not shown). When adjusted for counts of survived eggs in the control bags, a mean of 3% of the eggs placed in the five experimental excreta heaps were alive after 88 days (range 9 - 1%), 1% after 103 days (range 2 - <1%) and <1% after 117 days of storage. Thus, similar overall die-off times were seen for eggs despite some initial differences in the die-off rates. This showed a slower die-off rate for the excreta heaps with 0% lime and a more rapid initial egg die-off for the heaps with the highest pH. As seen in Figure 1, there was no difference in the die-off time between the heaps with high pH and those with a low pH. Interestingly, it was seen that even though lime was added to the heaps to obtain increased alkaline conditions with initial pH values ranging between 9.4 to 11.6, the variation in pH values after 133 days of storage narrowed to less than one pH unit (pH 8.6 - pH 9.5). The survival rate of controls was 63-70% throughout the experiment, with no difference between the control eggs inside the bags and the ones placed in petri dishes (results not shown).

**Figure 2:**
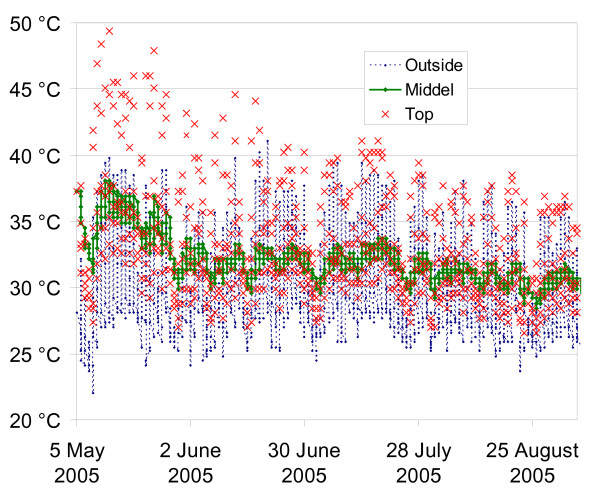
**The Temperature development in the top and the bottom of the heap with no lime added, plot together with the ambient temperature**.

There was no difference in the total die-off time between eggs in the tea bags inserted in the top and bottom of the excreta heap with 0% lime. Eggs in bags placed in the top of the heap had a 27% survival after 75 days as against 26% of eggs placed in the bottom. After 88 days, these rates were 9% versus 7%, respectively, with none of the eggs placed at both locations found viable after 103 days. However, when the eggs in the top and bottom were compaired to the once placed in the middle a slower die-off rate was observed, in the middle a 27% survival was reached after only 46 days storage which was 30 days prior to the date when the same die-off was reached in the bottom and top samples. These result suggest that the die-off rates for the helminth eggs were not significantly increased by the elevated temperatures registered in the top of the heap, which reached a maximum of 50.4°C, with simultaneous temperatures of 38.0°C in the middle and 35.3°C in the bottom of the heap (Figure 2).

Figure 2 shows the temperature development in the top versus the middle of the excreta heap and the ambient temperature as measured every four hours during the 120-day duration of the experiment. Except for the initial three weeks, the temperature development in the heaps followed the same pattern of the ambient temperature measured with the outdoor temperature logger, suggesting that a very limited thermophilic composting took place during the experiment. During the first three weeks, the average temperature in the bottom of the heap was 4°C higher than the average ambient temperature. Figure 2 shows large temperature variations inside the top of the heaps. Over a 24-hour period during the summer months, a variation of up to 18°C degrees was recorded (32-50°C) and in the cooler autumn months the variation was limited to 5-10°C with a minimum temperature of 26°C.

There was an increase in moisture content, from between 40-50% to 60-70%, in the heaps during the 120 days. That was most likely due to rainwater penetration of the tarpaulin used to cover the compost heaps.

**Figure 3:**
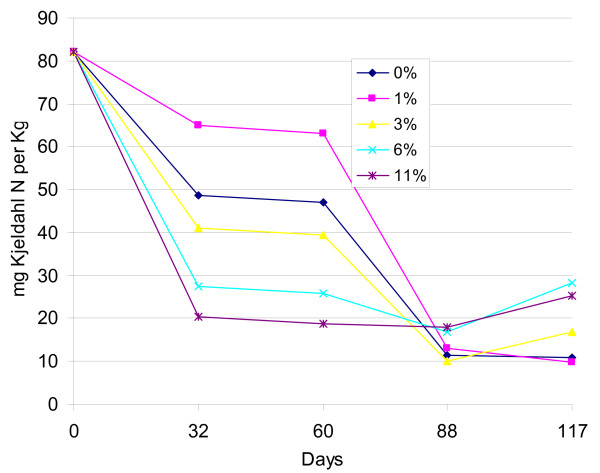
**Temporal development of Kjeldahl nitrogen (NH_3 _and NH_4_^+^) concentrations in excreta heaps with different lime content (%)**.

**Figure 4:**
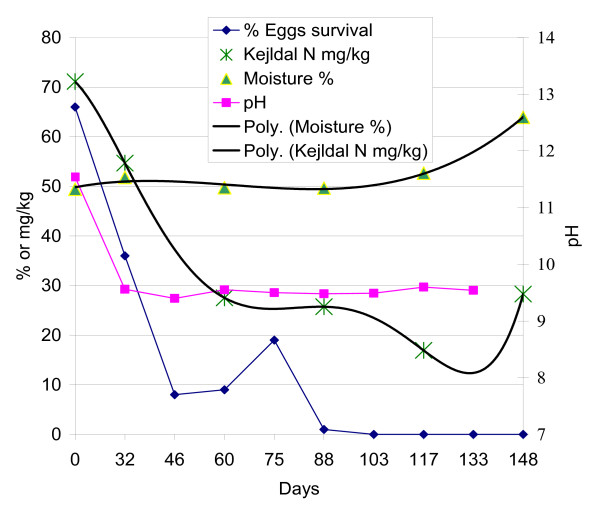
**Changes in the physical, chemical, and microbiological parameters in the heap amended with 3% lime**. A polynomial trend line is superimposed over the nitrogen and moisture measurements.

The average concentration of total nitrogen in the heaps was 97 mg N per kg excreta. The results presented in Figure 3 shows low pH (low percentage of lime) concentrations in the beginning of the experiment, which correlates with high organic nitrogen (NH_3 _and NH_4_^+^) concentrations. In comparison, heaps with a high pH (high percentage of lime) showed rapid changes in the content of organic nitrogen. These rapid changes are illustrated in Figure 4 where all the different parameters (pH, organic nitrogen, moisture and egg die-off) are listed for the heap with 3% lime. A polynomial trend line is added for the organic nitrogen and moisture analyses, as these were not done as frequently as the analysis of egg survival.

## Discussion

The main aim of this paper was to evaluate if the current practice of using Double Vault Composting (DVC) latrines in Vietnam provides hygienically safe excreta fertilizer following a three to four month storage period. When placing *Ascaris suum *eggs in heaps of human excreta with pH levels between 9.4-11.6, it took a maximum of 117 days to achieve a 99% die-off of the eggs, irrespective of the initial pH. This suggests that the Vietnamese farmers, through their normal storage and composting practices and under environmental conditions as described in this paper, could potentially produce hygienically safe human excreta for use as fertilizer in agriculture within the six month storage period stipulated in the national guidelines. Our findings also showed that the addition of lime at different concentrations to the excreta had a limited effect on pH development and egg survival. Previous research has also found a similar lack of correlation between the concentration of lime and *Ascaris *die-off in human excreta for pH below 12 [20]. However, other studies using sludge from waste water treatment plants report that the die-off time depends on pH and generally a pH >12 is recommended to obtain safe sludge for use as fertilizer [20-22].

Temperature increases in the excreta heaps due to microbiological processes were only seen to a limited extent and only within the first three weeks of storage. Thus, temperatures in the heaps were determined mainly by the ambient temperatures. Our findings are in accordance with other studies that showed little, if any, temperature development during storage (Figure 2). However, there were large variations on both the daily and weekly average temperatures. If we had used the midday temperatures measured inside the top of the heaps to estimate the die-off times, our results on egg survival would have been seriously overestimated. As an illustration of the problem of overestimation of egg survival, an average temperature over a 7-day period measured at 2:00 pm in the top part of the excreta heap would result in an estimated average temperature of 47°C, a temperature which according to the normal cited curve on *Ascaris *temperature versus die-off time would result in a 100% die-off after only five to six days of storage [23]. In contrast a similar exercise in the bottom of the heap (an exercise which is difficult to carry out inside the latrine vault) would result in an estimated average temperature of 35°C, which according to [23] gives a die-off time of more than a year. The increased temperatures in the top could be explained by a higher biological activity due to the supposed higher oxygen content close to the surface of the heap. However after examining the variation of the temperatures, it is more likely that the top data logger's proximity to the surface exposed it to the ambient temperatures. Therefore care must be taken when estimating the pathogen die-off time based on measured temperatures. To increase the safety margin in estimations of pathogen die-off time it is recommended that experiments use the night temperature as the daily average.

From the onset of the experiment, the die-off rate of *A. suum *eggs was highest in the heaps with the highest pH values. However, after approximately two months of storage this tendency disappeared and eggs in all heaps showed similar die-off rates until all eggs were found non-viable after 105 to 117 days. A similar trend was seen with the pH values with large variations in the beginning of the experiment of more than three pH units to variations of less than one pH unit after three months storage. Pecson et al. [22] also observed this "internal buffer" effect and argue that it is caused by ammonia volatilization with the ammonium ion, NH_4_^+^, being changed to ammonia, NH_3_, at alkaline conditions (pK_a _= 9.3 at 25°C). This could explain why we saw a large decrease in the organic nitrogen concentration as measured by the Kjeldahl N method (NH_4_^+ ^plus NH_3_) in the heaps with high pH values (Figure 3). The ammonia created under such alkaline conditions would volatilize and therefore lead to a lower pH. It does not seem to be the high pH itself that inactivates the *Ascaris *eggs, but the presence and negative impacts on egg survival of NH_3 _[22]. This could therefore explain why a fast die-off rate of eggs was seen in the beginning of the experiment in the heaps with the strongest decline in the content of organic nitrogen, i.e. free ammonia would have caused a kill-off effect before it volatilized. Our results further support this theory as illustrated by the individual heap where the curves for organic nitrogen, pH and *Ascaris *eggs die-off decreased simultaneously (Figure 4). The changes in the content of organic nitrogen had to be superimposed with a trend line to visualize the evaporation (Figure 4). Unfortunately we did not measure the Kjeldahl N before day 60 of the experiment. The concentration of NH_3 _and NH_4_^+ ^in our experiments was at maximum 82 mg N per kg excreta and to our surprise a similar effect was seen on egg survival when experiments were done with NH_3 _concentrations of 5000 mg/l in liquid sludge [22]. This demands further investigation under real-life conditions and concentrations of NH_3_, especially on how the rate of egg survival is affected during the first critical days/weeks of the contact between the eggs and NH_3_.

*A. suum *eggs are commonly used as a model organism for *A. lumbricoides*, the roundworm that often infect humans in less developed countries [24]. For survival and viability studies, eggs of *A. suum *are usually collected directly from the uterus of adult female worms. However, when excreted from the pig or human hosts, *A. suum *and *A. lumbricoides *eggs have been exposed to the gut environment and then to the external environment, i.e. in the pig sty and latrine. The maturation that the eggs undergo due to the exposure to the external environment is likely to make the eggs more resistant to environmental stress than eggs that have been removed directly from the uterus of adult female worms. Although it is preferable to use eggs for survival experiments that are recovered directly from faeces, this is rarely done because a heavy workload and a relatively low number of eggs are obtained compared with collecting eggs from adult female worms. Differences in the survival of eggs of different origin should be taken into consideration when evaluating and comparing results from studies on egg viability. As eggs collected from the uterus of adult worm were used in our study, it is likely that the viability of eggs was underestimated. There is an urgent need to establish how different development stages of eggs, their collection procedures, and their previous external environmental exposures impact the viability of *A*. *suum *eggs and its suitability as an indicator organism.

If our study results are evaluated on the basis of our previous findings, that a Vietnamese farmer would normally only store excreta for three to four months, which is the time period between the application of excreta-based fertilizers in the field [13], it is possible to estimate the rate of reduction of viable helminth eggs in human excreta used by farmers for crop fertilization. Presuppose that the vaults are used in three month intervals, i.e. three months for filling the vaults and three months storage under similar environmental conditions [13], and that there is a 97% die-off after three months and a >99% egg die-off after four months storage as found in the present study. Then only stored excreta produced in the last month of filling the vault may contain viable eggs; and of these eggs more than 97% will no longer be viable. This suggests that an average non-urine separating DVC latrine would reduce the number of viable *A. suum *eggs by more than 99% of the initial concentration after just three months of storage. From a public health point of view, our results suggest that the application of excreta that has been stored in DVC latrines to fields might only play a minor role in the overall transmission of helminth infections in Vietnamese households in rural areas, and the major source for the high prevalence of helminth infections could be found elsewhere, like in small children's indiscriminate defecation pattern outside the latrines as we observed in the area. However, these findings apply only to non-urine separation latrines. If the urine is separated, which is also a common practise in Vietnam, then longer storage times are probably needed since the egg die-off will not benefit from the impact of NH_3 _and NH_4_^+ ^in the urine. Further studies are needed to document to what extend urine separation may increase the survival rate of helminth eggs in stored excreta.

In order to quantify the risk to the household using human excreta as fertilizer composted inside the DVC, it is necessary to compare the health risk from the composted human excreta to other obvious transmission routes, like children defecating around the household premises etc. Given that a properly operated DVC can reduce helminth egg viability by more than 99%, it is a precondition that the users do not separate urine and faeces and the farmers operate the DVC properly and only empty one pit at a time and leave the other one untouched.

One limitation of our study was that the excreta were well-mixed, or at least better-mixed than excreta that are typically found in a DVC as a result of the hour-long mixing by shovel. This mixing will more evenly distribute the lime or ash within the excreta and prevent pockets of unmixed excreta that are not exposed to elevated pH. This effect would be expected to increase the inactivation rate in our experiment, as compared to the conditions in a real latrine vault. Therefore our results are not directly transferable to DVC latrines unless they are stirred on a regular basis.

A recent study concluded that the high prevalence of helminth infections in Vietnam are not related to latrine coverage in the population [6]. At a first glance, this study supports our conclusion; however, the study involved a population where 79% used a single vault latrine and 10% a DVC. The authors did not stratify between the two latrine types and based their analysis on the farmers' own answer to the question "if they used the excreta as fertilizer". Not surprisingly, only 17% replied that they used excreta in the fields [6]. This response could be related to the illegal practise of using vault latrines that do not allow stored excreta to be separated from fresh excreta. This also could be the reason why the authors were unable to link the use of fresh excreta from the single pit latrines to the high infection rate in the population. In our study, a similar tendency of significant underreporting was experienced when farmers were questioned about potentially illegal practises [13]. Therefore such questions should be avoided or critically evaluated via triangulation of information techniques.

## Conclusion

Under the climatic conditions existing in the summer months in northern Vietnam, the non-urine diverted Vietnamese Double Vault Composting latrine (DVC) could potentially provide human excreta that is safe to use as fertilizer in agriculture following a storage period of three to four months, if the latrine are stirred on a regular basis. These results are not necessary valid under the colder conditions in the winter months. Different concentrations of lime to increase the pH were not associated with an increased die-off of *A. suum *eggs. Free ammonia (NH_3_) is likely to impose a significant negative impact on the survival of *Ascaris *eggs. If storage times of only three to four months are possible, e.g. because farmers would need to empty the vaults after such short storage, then use of urine diverting latrines should not be recommended. Thus, the non-urine diverted DVC represents a feasible option for farmers in northern Vietnam providing them with a hygienic quality of human excreta that can be applied in fields with significantly reduced risks for helminth infections as compared to the direct use from single vault latrines.

## List of abbreviations

N: Nitrogen; NH_3_: Amonia; NH_4_^+^: Amonium.

## Competing interests

The authors declare that they have no competing interests.

## Authors' contributions

PKMJ and PDP: Conceived the idea, designed the study and carried out the research in the field.

LTK, FK and AD participated in its design and coordination and helped to draft the manuscript.

All authors read and approved the final manuscript.
